# Antagonistic interplay between hypocretin and leptin in the lateral hypothalamus regulates stress responses

**DOI:** 10.1038/ncomms7266

**Published:** 2015-02-19

**Authors:** Patricia Bonnavion, Alexander C. Jackson, Matthew E. Carter, Luis de Lecea

**Affiliations:** 1 Department of Psychiatry and Behavioral Sciences, Stanford University, 1201 Welch Road , Stanford, California 94305, USA; 2 Laboratory of Neurophysiology, Université Libre de Bruxelles (ULB)-UNI, 1070 Brussels, Belgium; 3 Department of Cellular and Molecular Pharmacology, University of California, San Francisco, California 94143, USA; 4 Department of Physiology and Neurobiology, University of Connecticut, Storrs, Connecticut 06269, USA; 5 Department of Biology, Williams College, Williamstown, Massachusetts 01267, USA

## Abstract

The hypothalamic–pituitary–adrenal (HPA) axis functions to coordinate behavioural and physiological responses to stress in a manner that depends on the behavioural state of the organism. However, the mechanisms through which arousal and metabolic states influence the HPA axis are poorly understood. Here using optogenetic approaches in mice, we show that neurons that produce hypocretin (Hcrt)/orexin in the lateral hypothalamic area (LHA) regulate corticosterone release and a variety of behaviours and physiological hallmarks of the stress response. Interestingly, we found that Hcrt neuronal activity and Hcrt-mediated stress responses were inhibited by the satiety hormone leptin, which acts, in part, through a network of leptin-sensitive neurons in the LHA. These data demonstrate how peripheral metabolic signals interact with hypothalamic neurons to coordinate stress and arousal and suggest one mechanism through which hyperarousal or altered metabolic states may be linked with abnormal stress responses.

## Abstract (web summary)

The hypothalamic-pituitary-adrenal axis coordinates behavioral and physiological responses to stress but the mechanisms are poorly understood. Here, the authors show that neurons that produce hypocretin/orexin in the lateral hypothalamic area regulate corticosterone release and a variety of behaviors related to the stress response.

## Graphical abstract

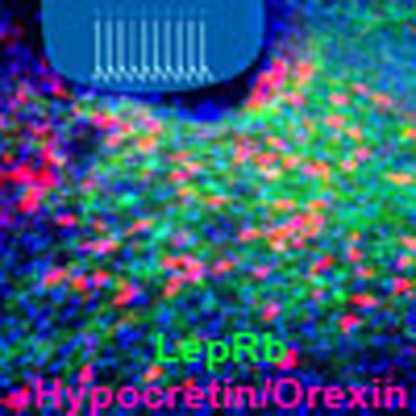

Stress is commonly defined as a state of threatened homoeostasis. The principal effectors of the stress response are localized in the paraventricular nucleus (PVN) of the hypothalamus, the anterior lobe of the pituitary gland and the adrenal gland, collectively referred to as the hypothalamic–pituitary–adrenal (HPA) axis. In response to stress, neuroendocrine pathways regulated by the HPA axis initiate a repertoire of physiological processes that culminate in the release of glucocorticoid hormones from the adrenal cortex. Aberrant activation of the HPA axis is a key feature of numerous psychiatric disorders and chronic metabolic illnesses^1^. Despite considerable research^2^,^3^,^4^, the central mechanisms that drive adaptive changes in HPA axis activity in response to metabolic challenges remain poorly characterized.

Neurons containing hypocretin peptide (Hcrt), also called orexin, are involved in the central regulation of arousal and energy balance, and many of their features indicate that the Hcrt system can modulate the intensity of the HPA axis’ response to stress^5^,^6^. Indeed, Hcrt neurons make reciprocal excitatory connections with corticotropin-releasing factor–containing neurons of the hypothalamic PVN, which are key actuators in the initiation of central stress responses^6^,^7^. Hcrt neurons also exhibit various firing profiles that are correlated with states of enhanced arousal or increased vigilance^8^,^9^. Accordingly, central administration of Hcrt stimulates the release of stress hormones, such as adrenocorticotropic hormone (ACTH) and corticosterone^6^,^10^,^11^,^12^,^13^,^14^, while Hcrt receptor antagonism attenuates stressor-induced increases in ACTH secretion^15^. Furthermore, Hcrt knockout animals have reduced flight-or-fight responses^16^. Imaging of c-Fos activity shows that Hcrt neurons are highly responsive to stress-related stimuli including electric footshocks, novel environments, restraint stress, hypercapnia or food deprivation^6^,^11^. These latter studies suggest that Hcrt neurons are capable of integrating a multitude of stress-related inputs, both central and peripheral, and are critical modulators and/or actuators in the neural circuitry of stress.

Among the numerous modulators of Hcrt neuronal activity^17^, leptin is particularly well-positioned to convey information about metabolic status to the Hcrt system^18^. In rodents, leptin was shown to inhibit the HPA axis during acute restraint stress, independent of its well-established role in satiety and energy intake^19^. Furthermore, defects in leptin signalling are associated with HPA axis hyperactivation and hypercorticosteronemia^20^,^21^. Most physiological functions of leptin are mediated centrally through leptin-responsive neurons expressing the long isoform of leptin receptor (LepRb) distributed throughout the hypothalamus, and most predominantly in the arcuate nucleus^18^,^22^,^23^,^24^. However, leptin may also regulate energy homoeostasis and motivated behaviour through another population of LepRb-expressing neurons, intermingled with Hcrt neurons in the lateral hypothalamic area (LHA)^22^,^23^,^24^,^25^. Anatomically, these LHA LepRb neurons appear to be largely GABAergic^25^ and to project onto a population of neighbouring Hcrt neurons^26^. However, how leptin modulates leptin-sensitive neurons in the LHA and affects Hcrt neuronal activity remains unclear.

Here we examine whether selective activation of Hcrt neurons is sufficient to initiate stress responses, including HPA axis activation, and investigate the context in which Hcrt neurons exert their influence on physiological and behavioural features of stress responses. We also examine the circuit-level mechanisms underlying the tuning of Hcrt neuron activity by leptin within the LHA and its consequences on HPA axis activation. Our results suggest that selective activation of Hcrt neurons is sufficient to drive stress responses, including HPA axis activation, and that leptin, in turn, attenuates HPA axis activation. This inhibition occurs, in part, through a network of LepRb-expressing inhibitory neurons, which suppress HPA axis activation mediated by the Hcrt system.

## Results

### Photostimulation of Hcrt neurons increases HPA axis activity

**Figure 1:**
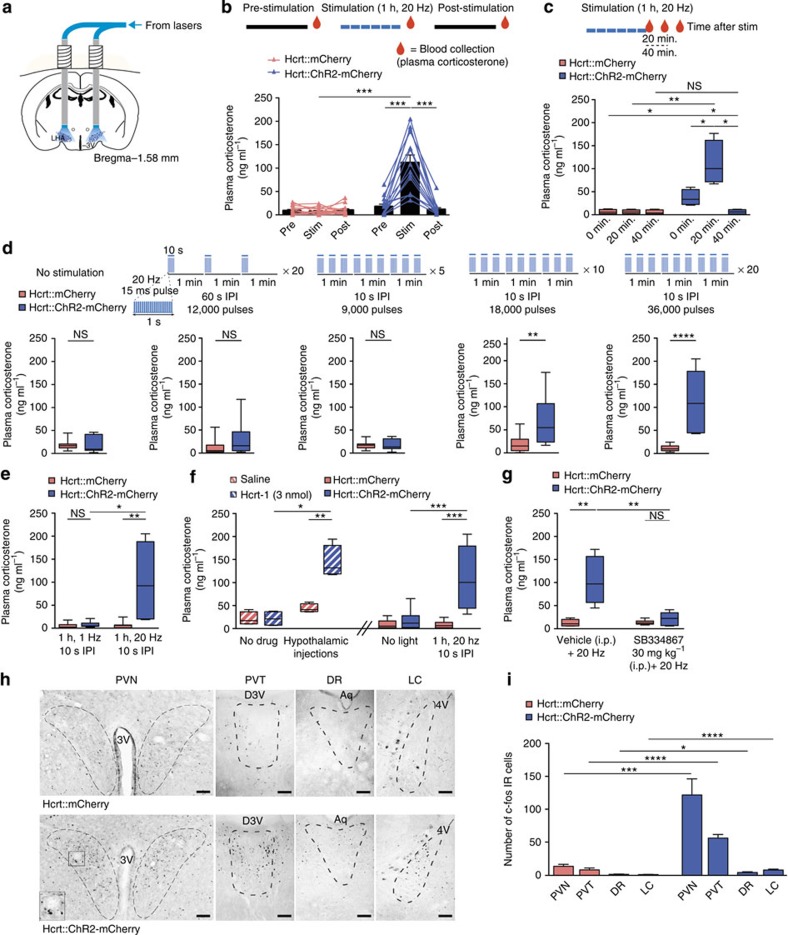
*In vivo* photostimulation of Hcrt neurons activates the HPA axis. (**a**) Experimental preparation. 3 V, third ventricle; fx, fornix. (**b**) Photostimulation of Hcrt neurons elevates plasma corticosterone in Hcrt::ChR2–mCherry mice (blue, *n*=14), but not in Hcrt::mCherry control mice (red, *n*=14). Triangles connected by lines represent values for individual mice. ^**‡**^*P*<0.0001; ^**§**^*P*<0.0001. (**c**) Time course of corticosterone secretion after 20 Hz photostimulation in ChR2 (blue, *n*=4) and control mice (red, *n*=4). ^**‡**^*P*<0.001; ^**§**^*P*<0.05. (**d**) Different modes of 20 Hz photostimulation using increased number of light pulses and reduced inter-pulse intervals (IPI) boost corticosterone levels in ChR2 mice (blue, *n*=11) compared with controls (red, *n*=10). ^**‡**^*P*<0.01; ^**§**^*P*<0.001. (**e**) Hcrt photostimulation increases corticosterone in a frequency-dependent manner (blue: Hcrt::ChR2–mCherry, *n*=7; and red: Hcrt::mCherry controls, *n*=7). ^**‡**^*P*<0.05. (**f**) Intrahypothalamic infusion of Hcrt-1 (dashed blue/white, *n*=4) elevates corticorticosterone levels compared with circadian baseline and saline-treated mice (dashed red/white, *n*=4) similar to Hcrt photostimulation. (**g**) Systemic injection of Hcrt antagonist SB-334867 (30 mg kg^−1^) prevents Hcrt-evoked increase in corticosterone concentrations (blue: Hcrt::ChR2–mCherry, *n*=6; red: Hcrt::mCherry controls, *n*=6). ^**‡**^*P*<0.05. (**h**) Representative photomicrographs of hypothalamic (PVN) and thalamic (PVT) paraventricular nuclei, dorsal raphe (DR) and locus cœruleus (LC) sections (delimited in dashed areas) stained for c-Fos from ChR2 and control mice. Scale bar, 100 μm. (**i**) Hcrt photostimulation increases the number of c-Fos immunoreactive (IR) neurons in the PVN, PVT, DR and LC of ChR2 (blue, *n*=7) compared with controls (red, *n*=7). ^‡^Two-way repeated measures ANOVA between viral transduction and stimulation condition, time intervals post-stimulation or pharmacological conditions. ^**§**^One-way repeated measures ANOVA followed by Tukey’s *post-hoc* test. NS, not significant (*P*>0.05), *P<0.05, **P<0.01, ***P<0.001, ****P<0.0001 (two-tailed Student’s *t*-test). Bars in **b** and **h** represent mean±s.e.m. In **c**–**f**, centre lines show the median, boxes extend from 25th to 75th percentiles, whiskers indicate minimum and maximum values.

To determine whether activation of the Hcrt system is sufficient to drive stress behaviours, we examined the effects of selective optogenetic control of Hcrt neurons on stress-related physiological parameters and behaviour. We first examined the effects of photostimulating ChR2-expressing Hcrt neurons on plasma corticosterone secretion as a readout of HPA axis activation in freely moving mice. We selectively transduced Hcrt neurons with lentivirus expressing Hcrt::ChR2–mCherry or Hcrt::mCherry and systematically measured plasma corticosterone concentrations in response to bilateral LHA photostimulation (Fig. 1a,b), as described in ref. 27. Extended phasic high-frequency photostimulation of Hcrt neurons (10-s pulse trains at 20 Hz, delivered three times per minute over 1 h) in freely moving ChR2 mice resulted in elevated plasma corticosterone concentrations compared with controls (*P*<0.0001, two-way repeated-measures analysis of variance (RM ANOVA), *n*=14 in each group; *P*<0.0001, one-way RM ANOVA in Hcrt::ChR2–mCherry mice; Fig. 1b–d). This reversible increase in plasma corticosterone is maximal when blood is collected and analyzed with a 20-min latency post-photostimulation (*P*=0.0002, two-way RM ANOVA, *n*=4 in each group; *P*=0.0193, one-way RM ANOVA in Hcrt::ChR2–mCherry mice; Fig. 1c). We tested several stimulation paradigms varying in duration and frequency to further examine the dynamics of HPA axis activity in response to Hcrt neuron stimulation (Fig. 1d). Corticosterone levels depended on the number and frequency of light pulses applied to the Hcrt system. At least 18,000 light pulses or 30-min photostimulation at 20 Hz with 10 s pulse and 10 s inter-pulse intervals (IPI) were necessary to increase corticosterone concentration in ChR2 mice (*P*=0.0235, two-way RM ANOVA, *n*=11 Hcrt::ChR2–mCherry and *n*=10 Hcrt::mCherry; *P*=0.0002, one-way RM ANOVA in Hcrt::ChR2–mCherry mice; Fig. 1d). Shorter (15 min) or lower frequency photostimulation bouts (60 s IPI) or slower photostimulation (1 Hz, 10 s IPI) (Fig. 1e) evoked no change in plasma corticosterone (*P*=0.4972, unpaired two-tailed Student’s *t*-test, *n*=7 per group). Corticosterone levels in control animals were not affected by any of the photoillumination patterns tested (*P*=0.93, one-way RM ANOVA; Fig. 1d).

We then examined the specificity of persistently activating Hcrt neurons using 1 h stimulation at 20 Hz (10 s pulse with 10 s IPI), which greatly elevated plasma corticosterone levels. Given the slow time course of the response, it is possible that prolonged activation of Hcrt neurons favours the extrasynaptic volume transmission of Hcrt at target sites, including the PVN, thereby initiating stress responses. Therefore, we compared the effects of photostimulation with direct hypothalamic injection of Hcrt-1 peptide (Fig. 1f). Plasma corticosterone levels were similar in both conditions, suggesting that Hcrt peptidergic tone in the anterior hypothalamus, including the PVN, is sufficient to drive elevations in plasma corticosterone and mimic photostimulation of Hcrt neurons (Fig. 1f). Importantly, the effect of Hcrt neuron photostimulation was blocked by systemic administration of the Hcrt receptor antagonist SB-334867 (*P*=0.0215, two-way RM ANOVA, *n*=6 per group; Fig. 1g), demonstrating the necessity of Hcrt release associated with this photostimulation pattern. We first verified activation of Hcrt neurons by determining c-Fos co-expression. Photostimulation induced a fourfold increase in c-Fos expression in Hcrt::ChR2 neurons compared with controls (*P*<0.0001, unpaired two-tailed Student’s *t*-test, *n*=7 per group; Supplementary Fig. 1a,b). To further investigate hypothalamic and possible extra-hypothalamic mechanisms, we mapped the activation of Hcrt-targeted neurons and found significant elevation of c-Fos in known targets of the Hcrt system; that is, the hypothalamic PVN (*P*=0.0002), thalamic PVN (PVT, *P*<0.0001), dorsal raphe (DR, *P*=0.0127) and locus coeruleus (LC, *P*<0.0001) in mice transduced with ChR2 compared with controls (unpaired two-tailed Student’s *t*-test, *n*=6 per group; Fig. 1h,i). In addition, we tested the circadian dependency of Hcrt-mediated effects on corticosterone levels, as the activities of both the Hcrt system and HPA axis can oscillate during the light–dark cycle^2^,^28^,^29^. Stimulation of Hcrt neurons during the dark (active) period (*P*=0.0034, two-way RM ANOVA, *n*=10 Hcrt::ChR2–mCherry and *n*=11 Hcrt::mCherry; Supplementary Fig. 2), when sleep pressure is relatively low, reliably increased corticosterone concentrations to a similar extent as during the light period, suggesting that the effects of photostimulation are independent of circadian regulation.

### Persistent activation of Hcrt neurons elicits stress

**Figure 2:**
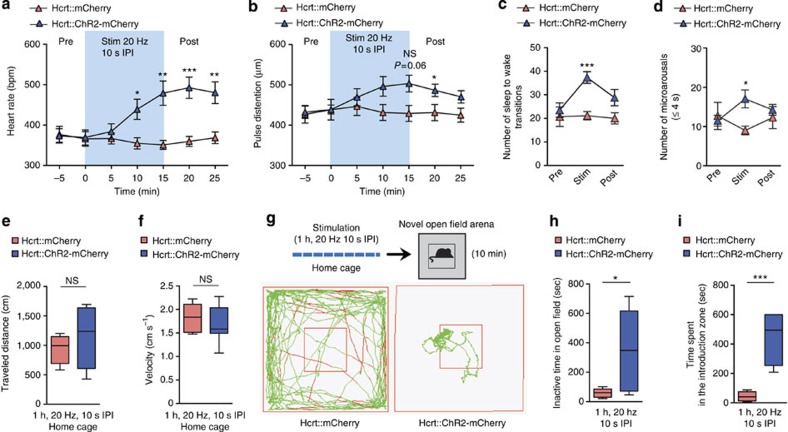
Long stimulation of Hcrt neurons induces a generalized state of stress. (**a**,**b**) Photostimulation at 20 Hz (10 s pulse, 10 s IPI) of Hcrt neurons in ChR2 mice (blue, *n*=6) causes an elevation of heart rate (**a**) and pulse distention (**b**), as an indicator of blood pressure, without affecting control mice (red, *n*=7). ***P<0.001 **P<0.01, *P<0.05 (**a**,**b**). (**c**,**d**) Persistent optogenetic stimulation of Hcrt neurons enhances sleep fragmentation characterized by increased sleep-to-wake transitions (**c**) and microarousal events (**d**). Sleep–wake parameters were recorded and averaged over 1 h before (Pre), during (Stim) and 1 h after (Post) Hcrt photostimulation in ChR2 (blue, *n*=9) and control mice (red, *n*=7). ***P<0.001 (**c**), *P<0.05 (**d**). (**e**–**f**) Hcrt photostimulation at 20 Hz does not modify home-cage locomotor activity. Bar graphs indicate total distance travelled (**e**) and average velocity (**f**) in ChR2 (blue, *n*=7) and control mice (red, *n*=5). *P*=0.37 (**e**) and *P*=0.54 (**f**), two-tailed unpaired Student’s *t*-test between transduced mice. (**g**–**i**) Hcrt-stimulated ChR2 mice failed to exhibit forward locomotor activity in new open field behavioural assay. (**g**) Diagrams show representative profile of mice activity in new open field. Green lines represent travel pattern and red lines indicate rapid activity (≥25 cm s^−1^). Red square indicates the introduction centre zone. (**h**,**i**) Stimulated ChR2 mice (blue, *n*=5) exhibit reduced field exploration measured by increased inactive time (**h**) and time spent in the introduction centre zone (**i**) compared with controls (red, *n*=6). *P<0.05 (**h**), and ***P<0.001 (**i**), two-tailed unpaired Student’s *t*-test between transduced mice. ^**‡**^Two-way repeated measures ANOVA between viral transduction and time epochs or stimulation condition. ^**§**^One-way repeated measures ANOVA followed by Tukey’s *post-hoc* test. NS, not significant (*P*≥0.05). Connected triangles in **a**–**d** represent mean±s.e.m. In **e**–**i**, centre lines show the median, boxes extend from 25th to 75th percentiles, whiskers indicate minimum and maximum values.

We next examined the consequences of phasic high-frequency photostimulation of Hcrt neurons on other hallmarks of the stress response, finding that 20 Hz photostimulation with 10 s IPI significantly elevated both heart rate (*P*<0.0001; Fig. 2a) and blood pressure (*P*=0.0006; Fig. 2b) in stimulated ChR2 but not in control mice (two-way RM ANOVA, *n*=6 Hcrt::ChR2–mCherry and *n*=7 Hcrt::mCherry). These observations are consistent with previous results using central administration of Hcrt peptide, which facilitates arousal by increasing both stress-related neuroendocrine function^10^ and sympathetic tone^15^.

We further investigated the consequences of initiating such stress responses on arousal state, and tested the ability of photostimulated mice to adapt in the face of an environmental challenge requiring increased arousal. Interestingly, long (1 h) photostimulation of Hcrt neurons at high frequency evoked a greater degree of sleep fragmentation in ChR2 mice, as evidenced by enhanced sleep-to-wakefulness transitions (*P*=0.0479, two-way RM ANOVA, *n*=9 Hcrt::ChR2–mCherry and *n*=7 Hcrt::mCherry; Fig. 2c) and increased microarousals during sleep (*P*=0.0289, two-way RM ANOVA; Fig. 2d), without modifying the total amount of wakefulness (*P*=0.50; Supplementary Fig. 3a) or total sleep (*P*=0.9902, two-way RM ANOVA; Supplementary Fig. 3b). In contrast, photostimulation in control animals did not affect sleep architecture (Fig. 2c,d, Supplementary Fig. 3a,b). We also examined spontaneous locomotion in response to 1 h photostimulation at 20 Hz in both groups. Stimulated ChR2 and control mice exhibited identical locomotor activity in their home cage, that is, total distance travelled (*P*=0.3717; Fig. 2e) and average velocity (*P*=0.5381, unpaired two-tailed Student’s *t*-test, *n*=7 Hcrt::ChR2–mCherry and *n*=5 Hcrt::mCherry; Fig. 2f). However, after extended Hcrt photostimulation, the same ChR2 mice exhibited increased inactive periods (*P*=0.0347; Fig. 2g,h) and stationary behaviour when placed in a novel open field (*P*=0.0007 unpaired two-tailed Student’s *t*-test, *n*=5 Hcrt::ChR2–mCherry and *n*=6 Hcrt::mCherry; Fig. 2g,i), with the overall activity of stimulated mice being similar to that of control animals (total distance travelled: 4,024±953.8 cm, Hcrt::ChR2 versus 4,923±385.7 cm, Hcrt::mCherry; *P*=0.41), and similar to the overall activity observed in the home cage. Such inhibitory behavioural responses to novel environment exposure are indicative of disrupted emotional reactivity. Taken together, these results suggest that extended stimulation of Hcrt neurons at high frequencies is capable of promoting a generalized state of stress.

### Food restriction modulates Hcrt influence on HPA axis

**Figure 3:**
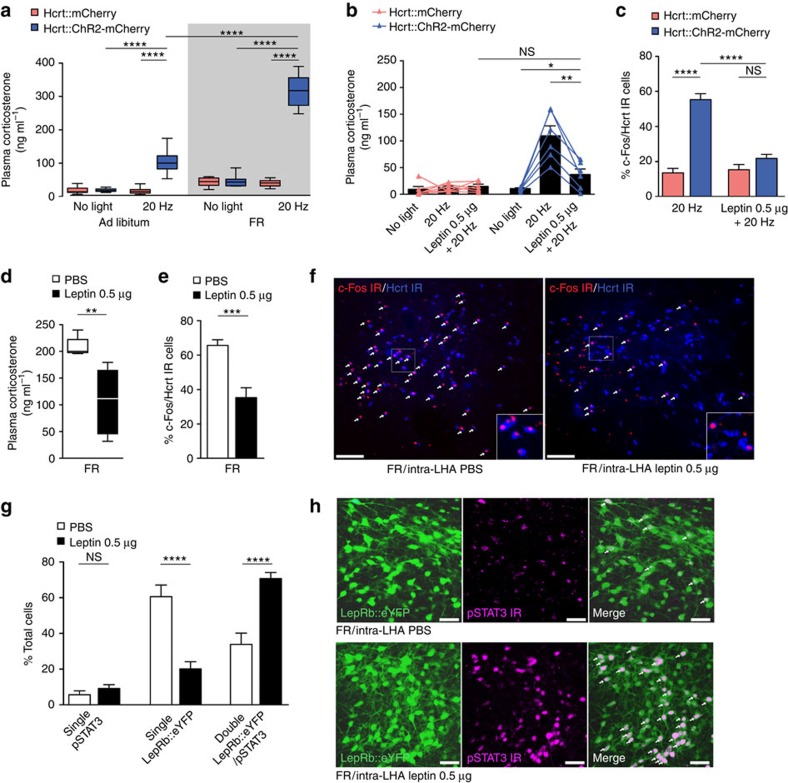
Leptin modulates Hcrt-evoked increase in plasma corticosterone. (**a**) Hcrt photostimulation (20 Hz, 1 h, 10 s IPI) following 7 days of food restriction (FR) enhances corticosterone increase in ChR2 mice (blue, *n*=10) compared with their stimulated fed state (*ad libitum*) or controls (red, *n*=10). ****P<0.0001. (**b**) LHA local injection of leptin (0.5 μg) suppresses Hcrt-evoked corticosterone increase in stimulated ChR2 mice (blue, *n*=6) compared with controls (red, *n*=6). *P<0.05, **P<0.01. Triangles represent corticosterone concentrations for individual mice. (**c**) Leptin inhibits c-Fos IR in stimulated Hcrt::ChR2 neurons (PBS: *n*=5, Hcrt::ChR2–mCherry/Hcrt::mCherry mice; leptin: *n*=6, Hcrt::ChR2–mCherry/Hcrt::mCherry mice). ****P<0.0001 (**d**) Wild-type mice receiving chronic bilateral injection of leptin (0.5 μg; black, *n*=5 FR) in the LHA showed reduced corticosterone concentrations following FR compared with PBS-treated mice (white, *n*=5 FR). **P<0.01. (**e**) LHA leptin injections suppress FR-induced c-Fos IR in Hcrt neurons (***P=0.001). Bar graphs quantify the percentage of double c-Fos/Hcrt IR neurons (leptin: black, *n*=5 FR; PBS: white, *n*=5 FR). (**f**) Representative photomicrographs of LHA sections co-stained for c-Fos (red) and Hcrt neurons (blue) from leptin and PBS-treated FR mice. White arrows identify double c-Fos/Hcrt IR neurons. Scale bar, 100 μm. (**g**) Bar graphs represent the percentage of pSTAT3-positive neurons and co-stained pSTAT3/LepRb–eYFP neurons in response to leptin and PBS treatment in FR mice (****P<0.0001). (**h**) Representative photomicrographs of LHA sections stained for pSTAT3 from PBS and leptin-treated LepRb–eYFP mice following chronic FR. White arrows identify LepRb–eYFP neurons (green) expressing pSTAT3 (magenta). Scale bar, 50 μm. ^**‡**^Two-way repeated measures ANOVA between viral transduction and feeding state conditions, or pharmacological conditions, or between pharmacological conditions and fed state. ^**§**^One-way repeated measures ANOVA followed by Tukey’s *post-hoc* test. NS, not significant (*P*≥0.05), *P<0.05, **P<0.01, ***P<0.001, ****P<0.0001 two-tailed Student’s *t*-test. Bars in **b**–**g** represent mean±s.e.m. In **a**, centre lines show the median, boxes extend from 25th to 75th percentiles, whiskers indicate minimum and maximum values.

Enhanced activity of the Hcrt system has been reported in states of negative energy balance^5^,^30^,^31^. Therefore, we examined whether Hcrt-induced HPA axis activation is modulated by environmental stressors, particularly those that influence metabolic needs. Restricted feeding or fasting modifies function of the HPA axis and causes chronic elevation of plasma corticosterone^32^. To examine how metabolic state influences the ability of Hcrt neurons to induce a stress response, we induced a state of negative energy balance by subjecting the mice to 7 consecutive days of food restriction (FR) to reach 85% of their initial body weight, and examined the effects of Hcrt photostimulation on plasma corticosterone levels (Fig. 3a). As expected, FR alone significantly increased basal corticosterone levels in mCherry control mice (*P*<0.0001, paired two-tailed Student’s *t*-test, *n*=10) and in unstimulated ChR2 mice (*P*=0.0002, paired two-tailed Student’s *t*-test, *n*=10). Interestingly, photostimulation of ChR2 mice further increased corticosterone concentrations 7.9-fold beyond that induced by FR alone and 3-fold beyond that induced by photostimulation of ChR2-expressing mice fed *ad libitum* (*P*<0.0001, two-way RM ANOVA; *P*<0.0001, one-way RM ANOVA). These data show that FR strongly facilitates Hcrt-mediated activation of the HPA axis.

### Leptin mediates HPA axis sensitization to Hcrt stimulation

One distinct effect of FR-induced HPA axis activation is a decrease in circulating leptin concentration^33^ that may in turn reduce feedback regulation onto the HPA axis^19^. Therefore, we assayed whether changes in leptin levels in the LHA can inhibit Hcrt neurons and prevent Hcrt-mediated activation of the HPA axis. First, we measured corticosterone concentration after 20 Hz Hcrt photostimulation with or without local infusion of leptin (0.5 μg) in the LHA (Fig. 3b,c). The observed Hcrt-evoked increase in corticosterone concentration was suppressed by local LHA leptin administration (*P*<0.0001, two-way RM ANOVA, *n*=6 per group; *P*<0.0001 and *P*=0.6214, one-way RM ANOVA in ChR2 and control mice, respectively; Fig. 3b). Furthermore, leptin suppressed c-Fos activation in photostimulated Hcrt-immunoreactive neurons (*P*<0.0001, unpaired two-tailed Student’s *t*-test, *n*=6 per group; Fig. 3c). These results suggest two possibilities that either leptin inhibits Hcrt cells and the optogenetic manipulation of Hcrt neurons is unable to overcome this inhibition, or that leptin excites a population of leptin-sensitive, LepRb-expressing, inhibitory GABAergic neurons in the LHA that provide inhibitory input onto Hcrt neurons. To distinguish between these possibilities, we examined the expression of c-Fos in Hcrt neurons, as a marker of neuronal activation, and the LepRb-dependent phosphorylated form of the transcription factor STAT3 (pSTAT3; refs 34, 35), to trace leptin signalling following FR. We injected transgenic LepRb::cre mice^23^,^36^ bilaterally with a control AAV–DIO::enhanced yellow fluorescent protein (eYFP) virus in the LHA, to allow us to visually identify neurons expressing LepRb. We then subjected these mice to chronic FR and locally administered either leptin (0.5 μg) or phosphate-buffered saline (PBS) daily within the LHA. We next analyzed corticosterone concentration and found that the corticosterone response to FR in LepRb::cre-eYFP mice was significantly reduced in leptin-treated mice compared with PBS-treated controls (*P*=0.0229, two-way RM ANOVA, *n*=5 per group; *P*=0.0074, leptin versus PBS-treated mice, unpaired two-tailed Student’s *t*-test; Fig. 3d). In addition, local leptin infusion in the LHA significantly decreased c-Fos activation in Hcrt neurons in response to FR (*P*=0.001, leptin versus PBS-treated mice, unpaired two-tailed Student’s *t*-test; Fig. 3d–f) and increased pSTAT3 immunoreactivity almost exclusively in LHA LepRb::eYFP neurons compared with PBS-treated controls (that is, 90.9±2.2% of total pSTAT3-expressing cells were LepRb-positive neurons; *P*<0.0001, leptin versus PBS-treated mice, unpaired two-tailed Student’s *t*-test; Fig. 3g,h). These results show that leptin-signalling through LepRb neurons in the LHA inhibits both Hcrt neurons and corticosterone release, thereby suppressing Hcrt’s effects on the HPA axis and could potentially prevent other Hcrt-mediated functions.

### LepRb GABAergic neurons innervate a subset of Hcrt neurons

**Figure 4:**
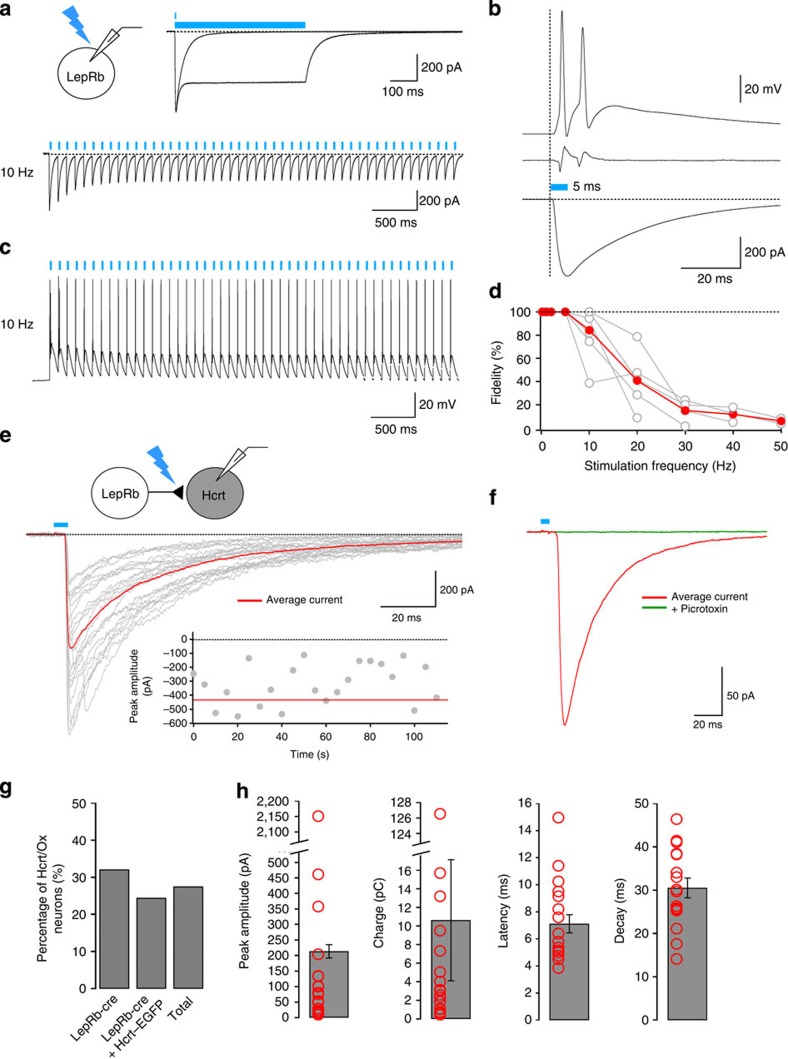
A subpopulation of LHA Hcrt neurons receives GABAergic innervation by LepRb neurons. (**a**) Representative voltage-clamp recording from a ChR2-expressing LepRb::cre neuron in the LHA, where ChR2-mediated photocurrents are evoked by blue-light pulses of varying duration (top: 5 and 500 ms) and frequency (bottom: 5 ms at 10 Hz). (**b**) Comparison of the time course of light-evoked responses (5 ms) in the same ChR2-expressing LepRb neuron; current-clamp (top), cell-attached patch (middle) and voltage-clamp (bottom). (**c**) Representative current-clamp recording of AP firing evoked by light pulses (10 Hz). (**d**) Fidelity of light-evoked firing over varying photostimulation frequencies (*n*=7). (**e**) Photostimulation evoked (5 ms) inhibitory postsynaptic currents (IPSCs) in an identified Hcrt neuron (individual currents in grey; average current in red), using a high-Cl internal solution at −60 mV in the presence of blockers of fast glutamatergic transmission NBQX and D-APV. Inset shows that >20 consecutive pulses delivered at 0.2 Hz showed no decrement in peak amplitude. (**f**) Average IPSC waveform before (red) and after (green) bath application of picrotoxin in a representative Hcrt neuron. (**g**) Proportion of Hcrt neurons exhibiting GABA IPSCs in each experimental condition: LepRb::Cre alone, LepRb::Cre/Hcrt::eGFP bi-transgenic and total. (**h**) Bar graphs showing peak amplitude (pA), charge (pC), latency (ms) and decay (ms) of light-evoked IPSCs.

Recent molecular and anatomical work suggests that GABAergic, LepRb-expressing neurons in the LHA^37^ make direct synaptic contacts with, and modulate gene expression in, a subpopulation of Hcrt neurons^26^. This mechanism may contribute to the observed reduction in c-Fos expression in Hcrt neurons following intra-LHA leptin infusions. To investigate the possibility that LepRb neurons provide functional, inhibitory synaptic inputs onto Hcrt neurons, we used an optogenetic approach (Fig. 4a–h) to simultaneously visualize Hcrt neurons and stimulate LepRb-expressing neurons for targeted patch-clamp recordings in hypothalamic slices. We used two strategies: (1) LepRb::cre transgenic mice injected with a mixture of AAV5–DIO–ChR2–eYFP and Lenti-Hcrt–mCherry viruses; and (2) Bi-transgenic LepRb::cre/Hcrt::enhanced green fluorescent protein (eGFP) mice injected with AAV5–DIO–ChR2–mCherry. In these experiments, ChR2 expression in LepRb neurons was found to be reliably dense within the LHA, but labelling sometimes extended beyond LHA boundaries. Recording from ChR2-expressing LepRb neurons in the LHA, we confirmed that blue-light photostimulation induced ChR2-mediated photocurrents (Fig. 4a) and reliably induced trains of action potentials, up to ~10 Hz (Fig. 4b–d). We then carried out whole-cell patch-clamp recordings from fluorescently labelled Hcrt neurons while photostimulating the cell bodies and/or terminals of LepRb-expressing neurons. In voltage-clamp recordings (in the presence of blockers of fast glutamatergic transmission), single pulses of light (5 ms) were capable of evoking rapid, inhibitory postsynaptic currents (IPSCs) in a subset of Hcrt neurons (Fig. 4e) that were blocked by the GABA_A_R antagonist picrotoxin (Fig. 4f). Overall, the percentage of labelled Hcrt neurons that exhibited photostimulation-induced IPSCs was 27.5% (Fig. 4g), with the two transgenic/viral strategies producing qualitatively similar results: 9/28 cells (32.1%) in LepRb-cre mice versus 10/41 cells (24.4%) in LepRb-cre/Hcrt–eGFP mice. Characteristics of light-evoked IPSCs (peak amplitude, charge, latency and decay) were pooled from both experimental conditions (Fig. 4h). These data demonstrate that LepRb-expressing neurons are capable of providing functional, GABA_A_R-mediated, inhibitory synaptic input onto a subpopulation of Hcrt neurons.

### LHA LepRb neurons downregulate HPA axis function

**Figure 5:**
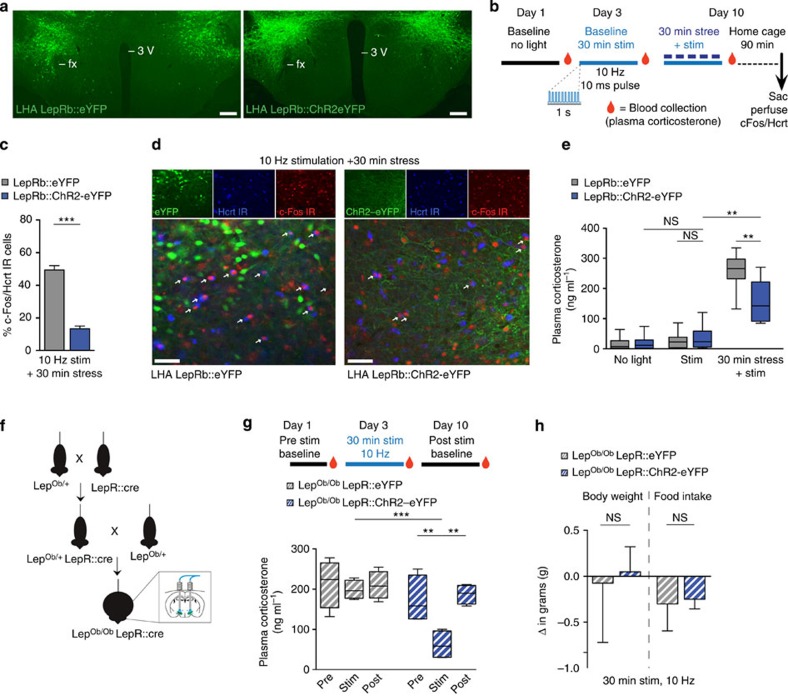
LHA LepRb neurons are crucial for the regulation of HPA axis activity. (**a**) Representative photomicrographs depicting viral eYFP and ChR2–eYFP expressions from LepRb::cre mice injected bilaterally into the LHA. Scale bar, 250 μm. (**b**) Experimental design. (**c**) Photostimulation of LHA LepRb neurons during restraint stress (RS) decreases stress-induced c-Fos IR in Hcrt neurons. Bar graphs quantify the percentage of double Hcrt/c-Fos IR neurons in LHA LepRb::ChR2–eYFP mice (blue, *n*=4) and LepRb::eYFP control (grey, *n*=6). ***P<0.001 two-tailed Student’s *t*-test between transduced mice. (**d**) Representative photomicrographs of LHA sections triple stained for LepRb (green), c-Fos (red) and Hcrt neurons (blue) from ChR2 and control mice following RS. White arrows identify double c-Fos/Hcrt IR neurons. Scale bar, 50 μm. (**e**) LepRb photostimulation reduces stress-induced corticosterone increase in ChR2 mice (blue, *n*=10), but not in controls (grey, *n*=10). ^**‡**^*P*<0.001; ^**§**^*P*<0.001. (**f**) Generation of LepRb::cre transgenic mice homozygous for the obese spontaneous mutation Lep^*Ob/Ob*^. (**g**) Photostimulation of LHA LepRb neurons is sufficient to correct hypercorticosteronemia induced by leptin deficiency in Lep^*Ob/Ob*^-LepRb::ChR2–eYFP mice (dashed white/blue, *n*=4) compared with Lep^Ob/Ob^-LepRb::eYFP controls (dashed white/grey, *n*=4). **P<0.01, ***P<0.001. (**h**) LHA LepRb photostimulation in obese^Ob/Ob^ mice does not alter body weight and food intake. Bar graphs represent the change in grams in body weight and food intake measured pre- and post-photostimulation of LHA LepRb neurons in Lep^Ob/Ob^-LepRb::ChR2 (*n*=4) and Lep^Ob/Ob^-LepRb controls (*n*=4). *P*=0.77 (body weight) and *P*=0.79 (food intake), two-tailed unpaired Student’s *t*-test between transduced mice. ^**‡**^Two-way repeated measures ANOVA between viral transduction and stress condition, or stimulation condition.^**§**^One-way repeated measures ANOVA followed by Tukey’s *post-hoc* test. NS, not significant (*P*≥0.05), *P<0.05, **P<0.01, ***P<0.001 two-tailed Student’s *t*-test. Bar graphs in **c** and **h** represent mean±s.e.m. In **e** and **g**, centre lines show the median, boxes extend from 25th to 75th percentiles, whiskers indicate minimum and maximum values. 3 V, third ventricle; fx, fornix.

**Figure 6:**
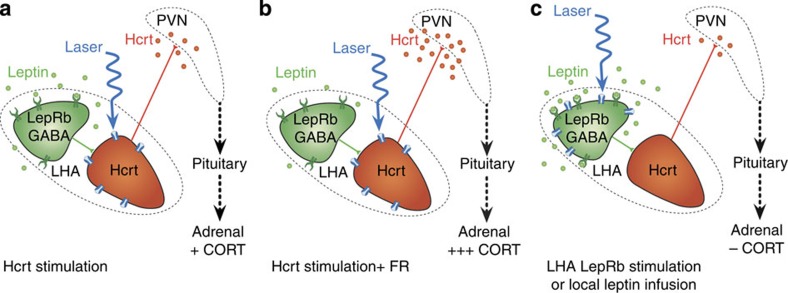
Hypothetical LHA circuit linking metabolic and stress signals. (**a**–**c**) Diagram representing a hypothetical model for a hypothalamic circuit that integrates metabolic state and stress responses through functional connections between LHA leptin-sensitive LepRb neurons, Hcrt cells and the HPA axis. We summarize here three conditions at different concentrations of circulating leptin: under baseline conditions, the interaction between leptin and hypocretin is able to set an appropriate stress response activating the HPA axis through the PVN (**a**); In **b**, negative energy balance state is associated with low leptin levels, which facilitates Hcrt-mediated activation of the HPA axis and corticosterone release. In contrast, high leptin concentrations (induced optogenetically or pharmacologically) suppress Hcrt-mediated responses on the HPA axis and decrease corticosterone release in response to stress (**c**).

To test the functional connectivity between LHA LepRb and Hcrt neurons and downstream modulation of the HPA axis *in vivo*, we photostimulated ChR2-expressing LHA LepRb neurons during restraint stress, a behavioural stress procedure that both activates the Hcrt system^7^ and elicits an increase in plasma corticosterone secretion^38^ (Fig. 5a,b). In all animals tested, eYFP expression was restricted to the LHA (Fig. 5a, Supplementary Fig. 4a). We restrained the mice for 30 min during continuous 10 Hz photostimulation of LHA LepRb neurons and first examined c-Fos expression in Hcrt neurons (Fig. 5b). Optogenetic stimulation in the LHA suppressed restraint-stress-mediated c-Fos expression in Hcrt neurons in LepRb::ChR2–eYFP but not in control eYFP mice (*P*<0.001, unpaired two-tailed Student’s *t*-test, *n*=4 LepRb::ChR2–eYFP and *n*=6 LepRb::eYFP; Fig. 5c,d), demonstrating that LHA LepRb neurons control responses of Hcrt neurons to acute stress (Fig. 6). In addition, we examined the amplitude of the neuroendocrine stress response under LHA LepRb activation. Photostimulation of LHA LepRb neurons during stress decreased plasma corticosterone concentrations by 37.1% (*P*=0.0004, two-way RM ANOVA, *n*=10 per group; *P*=0.0002, one-way RM ANOVA in LepRb::ChR2 mice; Fig. 5e). Histological examination of the brains following the experiments did not reveal any ascending projections from LHA LepRb::ChR2–eYFP neurons to the PVN (Supplementary Fig. 4b), indicating that LHA LepRb neurons inhibit the HPA axis indirectly (Fig. 6).

To further test the connection between activation of LHA LepRb neurons and modulation of HPA axis activity, we examined the effects of photostimulating LHA LepRb neurons under different states of HPA axis activation. We asked whether the activity of LHA LepRb neurons could normalize plasma corticosterone levels in an animal model of chronic HPA axis hyperactivity. Leptin-deficient obese (Lep^Ob/Ob^) mice exhibit chronic hypercorticosteronemia that can be corrected by systemic administration of leptin^21^. We explored the possibility that optogenetic stimulation of LHA LepRb neurons in Lep^Ob/Ob^ mice is sufficient to normalize hypercorticosteronemia. We crossed LepRb::cre transgenic mice with Lep^Ob/+^ mutant mice to generate Lep^Ob/Ob^-LepRb::cre mice (Fig. 5f). We found that acute photostimulation of LHA LepRb neurons (10 Hz, 30 min, 10 ms pulses) was sufficient to reduce and correct corticosterone levels in Lep^Ob/Ob^ mice (*P*=0.0314, two-way RM ANOVA, *n*=4 per group; *P*=0.0327, one-way RM ANOVA in Lep^Ob/Ob^-LepRb::ChR2 mice; Fig. 5g). These results demonstrate a novel role for LHA LepRb neurons in the central leptin-mediated inhibition of HPA axis activity. Our observations also identify one pathway that may contribute to the HPA axis dysfunction associated with leptin deficiency (Fig. 6).

## Discussion

Our study demonstrates a causal relationship between prolonged high-frequency activation of Hcrt neurons and stress. We show that *in vivo* optogenetic stimulation of Hcrt neurons induces a circadian-independent, reversible state of hypercorticosteronemia corresponding to increased HPA axis activity, which is associated with enhanced cardiovascular autonomic function, sleep fragmentation and disrupted exploratory behaviour. We also demonstrate that the Hcrt-evoked increase in corticosterone concentrations is influenced by metabolic state, notably in a leptin-dependent manner, as the local action of leptin within the LHA suppresses both Hcrt neuron excitability and corticosterone release (Fig. 6). Furthermore, we provide evidence that the inhibitory action of leptin on the Hcrt system is mediated, in part, by a subpopulation of LepRb-expressing, GABAergic neurons. Finally, we demonstrate that the selective activation of LepRb neurons in the LHA is sufficient to decrease stress-induced corticosterone release and normalize hypercorticosteronemia in leptin-deficient mutant mice.

Acute and semi-chronic optogenetic stimulation of Hcrt neurons has been shown to affect the probability of wakefulness^27^,^39^,^40^ and this increase in behavioural state transitions may alter the release of stress hormones. However, we describe here that shorter bouts of Hcrt neuron photostimulation (<15 min, or with 60 s IPI) or slower photostimulation (1 Hz) do not modify corticosterone secretion, suggesting that sustained changes in Hcrt neurotransmission and longer integration times by effector hypothalamic systems (for example, corticotropin-releasing factor neurons in the PVN) are necessary to elicit stress responses. Alternatively, direct or indirect activation of other extra-hypothalamic relay systems receiving dense Hcrt inputs, such as the PVT, DR or LC may contribute to the behavioural, neuroendocrine and autonomic responses to stress^41^,^42^,^43^. The long stimulation paradigm (1 h, 20 Hz, 10 s IPI) used in this study reveals a different timescale of Hcrt neuronal activation, likely elicited by extrasynaptic release over large areas innervated by Hcrt terminals, including the PVN (Fig. 6). However our observations cannot rule out that extra-hypothalamic mechanisms may occur, notably involving stress-related noradrenergic systems^43^,^44^,^45^,^46^.

Hcrt-mediated activation of the HPA axis is also linked to increased heart rate, enhanced sleep fragmention and disrupted exploratory behaviour when stimulated animals are subjected to a novel environment. Open field behavioural assays are commonly used to test both locomotor activity and emotionality in rodents. The purpose of these experiments was to test the reaction of stimulated animals to a stressful event such as exposure to novelty in an unfamiliar environment. Here the stimulated animals barely moved from the centre zone, showing stationary or freezing-like behaviours and decreased field exploration. These behaviours may be explained by Hcrt-mediated modulation of locomotor activity^5^,^47^. Indeed, sustained stimulation of Hcrt neurons can be expected to result in hyperlocomotion or increased activity in the open field. However, we did not observe any differences in general locomotor activity in either the open field or the home cage between stimulated and control mice. Thus, it is unlikely that phasic high-frequency photostimulation of Hcrt neurons induced locomotor alterations. It is also possible that altered emotional state led to disrupted (that is, reduced) open field behaviour. Our observations suggest that sustained activation of the Hcrt system facilitates a behavioural response to a novel environment characterized by the inhibition of exploration, and likely perceived as a stressful event. Further behavioural tests will be necessary to further explore this hypothesis.

One concern, as in most *in vivo* optogenetic studies, is whether the induced activation reflects physiological conditions and/or is the result of endogenous Hcrt neuronal firing associated with stress-inducing stimuli. The long photostimulation paradigm used was chosen based on *in vitro* and *in vivo* observations^8^,^9^,^48^,^49^,^50^. Previous *in vitro* observations showed the ability of Hcrt neurons to fire at a maximal frequency of 100 Hz in response to a single brief depolarization pulse^50^. Similarly, brief (1 s) continuous light illumination in ChR2-expressing Hcrt neurons evokes robust action potential trains with firing frequencies ranging from 8 to 25 Hz (ref. 27). *In vivo*, although the mean Hcrt neuron discharge rate in the wake state is reported to be ~3 Hz, with an instantaneous firing rate of 12 Hz (refs 8, 9, 49), the firing rate of these neurons can vary depending on behavioural state, reaching frequencies >10 Hz during high vigilance states, which are associated with increased muscle tone and/or electroencephalographic (EEG) desynchronization^9^. Indeed, Hcrt neurons displayed firing rates up to and above 15 Hz during active wake states^8^, particularly when subjected to an unpredictable sound stimulus^9^. Further *in vivo* recordings in the mouse LHA showed that Hcrt neurons are able to fire in high-frequency clusters (mean instantaneous frequency<50 Hz) at sleep-to-wake transitions^51^. Furthermore, recent work in brain slices demonstrated that optogenetic stimulation of Hcrt neurons at 20 Hz is capable of eliciting the release of both glutamate and Hcrt peptide over different timescales^52^,^53^. The firing rate of Hcrt neurons during states of stress and anxiety remain unknown. Interestingly, elevated Hcrt neurotransmission is associated with anxiety/panic attacks in humans and activation of the Hcrt system is necessary for the development of panic-like responses^54^. Based on these observations, and taking into account the modulatory nature of Hcrt discharge, which is positively correlated with enhanced cortical activation, we hypothesize that Hcrt neurons further increase their firing rate in response to stressors or during panic attacks. Perhaps such a hyperactive, overstimulated Hcrt system may thus reflect pathological conditions associated with HPA axis hyperactivation and anxiety states. Although enhanced Hcrt activity is hypothesized to promote increased motivational behaviour such as foraging and/or food seeking in food-restricted conditions or fasting^5^,^55^, our results suggest that dysfunction in Hcrt neuron excitability may instead favour a generalized state of stress or anxiety leading to maladaptive or pathological behaviour in the face of environmental challenges.

Evidence that metabolic signals directly modulate the activity of the Hcrt system and its role in stress derive from experiments in which calorie restriction enhances the activation of Hcrt neurons following social defeat stress^31^. We hypothesize that the decrease in circulating leptin concentrations resulting from chronic FR may contribute to this modulation. Indeed, leptin treatment prevents a fasting-induced increase in pre-pro-Hcrt messenger RNA^56^ and inhibits the firing of Hcrt neurons^5^. Furthermore, food deprivation is associated with increased excitatory synaptic inputs to Hcrt neurons, which is blocked by leptin and reversed by re-feeding^30^. These results suggest that a state of negative energy balance, induced by changes in central leptin signalling, may unmask a form of disinhibition of Hcrt neuronal activity that may, in turn, potentiate Hcrt-dependent activation of the HPA axis (Fig. 6).

Tracing experiments have shown that LHA LepRb neurons project directly onto a subpopulation of Hcrt neurons^26^,^57^ and negatively control the response of Hcrt neurons to fasting^57^. Also, previous studies in the LHA revealed pSTAT3 immunoreactivity in GAD67-expressing neurons, suggesting that LHA LepRb neurons are largely GABAergic^37^. In agreement with these results, we show that a subpopulation of LepRb neurons is capable of releasing GABA directly onto a subpopulation (27.5%) of Hcrt neurons. Interestingly, this result broadly coincides with the proportion of Hcrt neurons found to be connected with LepRb::cre neurons using trans-synaptic tracing methods^26^. However, in both cases, we cannot discount the possibility that LepRb neurons in nearby hypothalamic regions, outside the LHA, also provide input to Hcrt neurons. Adding to the complexity, leptin has been shown to have heterogeneous effects on the excitability of LHA LepRb neurons^37^,^57^. Furthermore, neurotensin-expressing neurons within the LHA, a population that overlaps with LHA LepRb neurons^57^, were recently shown to extend glutamatergic projections to dopaminergic neurons in the ventral tegmental area^58^. More recent work has shown that pharmacogenetic activation of LHA neurotensin neurons in brain slices results in the inhibition of Hcrt neurons, and that leptin indirectly inhibits Hcrt neurons through a GABA-independent mechanism involving presynaptic inhibition of excitatory inputs and activation of K(ATP) channels^25^. Intriguingly, this is thought to occur through release of the neuropeptide galanin^25^, which was shown to be expressed in another population of LHA neurons that overlaps with neurotensin^59^. The inhibition of Hcrt firing by leptin is consistent with previous results from acutely dissociated neurons^5^, a paradoxical effect given that Hcrt neurons do not appear to express the LepRb receptor^25^,^26^,^37^. These contrasting observations underscore the heterogeneous nature of such leptin-sensitive neurons and the diverse mechanisms through which leptin may tune the excitability of Hcrt neurons, and indicate that much remains to be learned about the functional significance of this diversity. Nevertheless, our *in vivo* experiments suggest that the dominant effect of both local LHA leptin infusion and optogenetic activation of LHA LepRb-expressing neurons is a suppression of neuronal activity in Hcrt neurons.

We show here that *in vivo* photostimulation of LHA LepRb neurons at a frequency of 10 Hz consistently reduces neuroendocrine stress responses under two conditions of HPA axis hyperactivity. The photostimulation paradigm was chosen based on previous *in vitro* and *in vivo* recordings obtained from posterior lateral hypothalamic neurons and non-Hcrt/non-MCH neurons of the LHA^49^,^60^,^61^,^62^. To date, *in vivo* recordings from identified LHA LepRb neurons have not been reported. As mentioned above, LepRb neurons are distinct from Hcrt- and MCH-containing neurons, and represent, at least in part, a subpopulation of LHA GABAergic neurons^25^,^26^,^37^. We thus applied a photostimulation paradigm consistent with previous observations of the firing rate of LHA non-Hcrt/non-MCH GABAergic neurons. *In vitro,* whole-cell current-clamp recordings of these neurons show a spontanous firing rate between 9 and 12 Hz depending on the circadian time^62^. *In vivo*, LHA non-Hcrt/non-MCH GABAergic neurons, the activity of which can be maximal when animals are sleeping, fire from 7 to 13 Hz across vigilance states^61^. In addition, another subpopulation of these neurons, independent of sleep/wake states, exhibit various firing frequencies varying from 10 to 17 Hz (refs 49, 60). We found that activation of LHA LepRb neurons with continuous 10 Hz photostimulation is capable of decreasing the acute HPA axis response to restraint stress and also reverses hypercorticosteronemia associated with leptin deficiency. This effect is not mediated through direct projections of LHA LepRb neurons to the hypothalamic PVN but likely involves several nodes targeting the PVN. Indeed, LHA LepRb neuron-mediated inhibition of the HPA axis under restraint stress is associated with suppression of the Hcrt system. In addition, we observed that similar photostimulation of LHA LepRb neurons also normalizes plasma corticosterone concentrations in Lep^Ob/Ob^ mice, a model of chronic hypercorticosteronemia in which both Hcrt and hypothalamic neuropeptide Y system are dysregulated^21^,^63^,^64^ and could therefore contribute to HPA axis hyperactivity^18^,^65^,^66^.

Interestingly, there is an inverse correlation between the circadian rhythms of plasma leptin and HPA axis activity, which also correlates with the circadian regulation of Hcrt neuronal activity and Hcrt messenger RNA levels^8^,^9^,^20^,^67^. Indeed, the link between circadian oscillation and Hcrt activity may be direct, as a recent report shows that Hcrt can suppress activity of suprachiasmatic nucleus neurons^68^. However, it is unclear whether the firing rate of LHA LepRb neurons is subject to circadian and/or behavioural state control. Some LHA non-Hcrt/non-MCH GABAergic neurons show a state-dependent pattern of activity with maximal firing during sleep but these neurons also remain active during the wake state^61^. Another population of non-Hcrt/non-MCH GABAergic neurons has been shown to fire in a non-state-dependent manner. Although plasma leptin exhibits a circadian profile^20^, LepRb neurons in the LHA appear to be regulated in a different manner, independent of circadian control by the suprachiasmatic nucleus. Our results are consistent with the notion that a balance between the activity of Hcrt and leptin-sensitive neurons participates in the circadian regulation of HPA axis activity, but this hypothesis should be examined further with regard to the activity of LepRb neurons across the light–dark cycle *in vivo* and possible interactions downstream of the suprachiasmatic nucleus.

In summary, we describe a link between arousal, metabolism and stress responses at the cellular and circuit levels. Collectively, our study reveals an important role for Hcrt and leptin, acting through hypothalamic leptin-sensitive GABAergic neurons, and suggests that an imbalance between the activity of LepRb-expressing neurons and Hcrt neurons in the LHA could lead to disruptions in HPA axis activity, which may depend on behavioural context and energy balance.

## Methods

### Animals

All behavioural experiments conducted to target Hcrt neurons were done on C57BL/6 J wild-type male mice obtained at ~7 weeks of age from the Jackson Laboratory. LepRb::IRES-cre knockin mice^69^ were kindly provided by Dr Martin G. Jr Myers (University of Michigan, Ann Arbor, MI, USA) and bred (>6 generations) onto a C57BL6/J background. LepRb::cre transgenic mice homozygous for the obese spontaneous mutation Lep^*Ob/Ob*^ were obtained by intercrossing heterozygous Lep^*Ob/+*^ mice (B6.V-Lep^*ob*^/J from the Jackson Laboratory, stock number 000632) with LepRb::cre mice. Mice were housed in individual Plexiglas recording chambers in custom-designed stainless-steel cabinets at constant temperature (22±1 °C), humidity (40–60%) and circadian cycle (12-h light–dark cycle, starting at 0900 hours). Food and water were available *ad libitum* except for chronic FR procedures (see Stress procedures). All experiments were performed in accordance with the guidelines described in the US National Institutes of Health Guide for the Care and Use of Laboratory Animals and were approved by the Institutional Animal Care and Use Committee of Stanford University.

### Virus preparation

We used high-titer (>10^10^ viral molecules per ml) *Hcrt::ChR2–mCherry* or *Hcrt::mCherry* control lentiviral vectors as described previously to target Hcrt neurons^27^ and double *loxP*-flanked inverted (DIO) AAV constructs to express ChR2–eYFP fusions and eYFP alone in LepRb::cre-expressing neurons. Cre-inducible recombinant AAV vectors carrying optogenetic transgenes, AAV–Ef1a–DIO–hChR2(H134R)–eYFP–WPRE–pA, AAV–Ef1a–DIO–hChR2(H134R)–mCherry–WPRE–pA and AAV–Ef1a–DIO–eYFP–WPRE–pA, were serotyped with AAV5 coat proteins and packaged by the viral vector core at the University of North Carolina. The final viral concentration was 4 × 10^12^ virus molecules per ml.

### Surgery

Mice were anaesthetised with a mixture of ketamine (80 mg kg^−1^) and xylazine (10 mg kg^−1^) injected intraperitoneally (i.p.), and placed on a small animal stereotactic frame (David Kopf Instruments). Recombinant Hcrt::ChR2–mCherry or control Hcrt::mCherry lentivirus (1 μl), or DIO–ChR2–eYFP or control DIO–eYFP AAV virus (0.2 μl; Fig. 5a) were bilaterally injected within the LHA (anteroposterior (AP), 1.5 mm; mediolateral (ML),±1.13 mm; dorsoventral (DV) 5.20 mm) through a 33-gauge injector cannula (Plastics One) using a syringe pump (World Precision Instruments) at a rate of 0.1 μl min^−1^. The injection cannula was left in place for 15 min following the injection and then slowly removed. After the viral injection, a 26-gauge bilateral cannula with 2 mm between individual cannulae and 5-mm shaft length was slowly lowered into the brain, placed above the LHA (AP, 1.5 mm; ML,±1.0 mm; DV, 4.7 mm), and affixed to the skull with C&B Metabond (Parkell) and dental acrylic. To allow time for recovery after surgery and viral expression, we housed animals for 2 to 3 weeks following injection in individual cages before any experiments were initiated. Optical fibres (200 μm, Thorlabs) were inserted into the implanted cannula guide, extending 0.3 mm below the guide, at least 3 days prior to the experimental procedures.

### *In vivo* photostimulation

All photostimulation experiments were conducted bilaterally and performed between 0900 and 1130 hours. The light sources were two diode-pumped 473 nm blue lasers (LaserGlow) controlled simultaneously by a Master-8 stimulator (AMPI) to generate light pulses of various durations (15 ms on Hcrt neurons and 10 ms on LepRb neurons) and frequencies (1 to 20 Hz for Hcrt neurons and 10 Hz for LepRb neurons). The power output was measured at the tip of the optical fibre with a light meter and preset at ~15 mW when the laser was activated in continuous mode. A maximum of four photostimulation sessions per animal was applied 7–10 days apart.

### Blood collection and plasma corticosterone analysis

Blood was sampled between 1000 and 1200 hours during light cycle or collected during the first 4 h of dark cycle under red lights by a tail-clip technique. Mice were placed on an unfamiliar surface and allowed to explore while holding the base of the tail. The distal ~1 mm of the tail was clipped and a heparinized microhematocrit capillary tube (Fisher Scientific) was used to collect ~40 μl sample within 2 min manipulation. Plasma was separated by centrifugation (5 min, 12,000 r.p.m.) and immediately stored at −80 °C until analyzed. Pre-stimulation and post-stimulation corticosterone baseline concentrations were analyzed 2 days before (pre) and 7 days after (post) the photostimulation experimental day (stim), at the corresponding circadian time. Once corticosterone time course was determined (Fig. 1c), blood was removed 20 min after the end of the photostimulation sessions or stress procedures. Plasma corticosterone (final dilution 1:40) was measured by a competitive immunoassay (EIA corticosterone kit, Enzo Life Sciences) following manufacturer’s protocol. The sensitivity threshold of the assay was 27 pg ml^−1^. At the time of blood removal and corticosterone assay, mice identification was unknown to the experimentator performing the tests.

### Locomotor monitoring

Home-cage mice locomotor activity was measured using the integrated modular platform SmartCage TM (AfaSci) equipped with infrared sensors that allow the platform to gather total distance travelled and average velocity data.

### Polysomnographic recording and analysis

After stereotaxic viral injection and cannula implantation, some Hcrt::ChR2–mCherry and Hcrt::mCherry mice were also implanted with a custom-made EEG/electromyographic (EMG) implant placed lateral to the cannula as previously described^27^,^39^. Mice were housed individually for at least 10 days after surgery and were then connected to commutators with flexible cables for habituation to the EEG/EMG recording conditions. Fibre-optic cables were inserted 3 days before the experiments and ran alongside the EEG/EMG connection cables. All sleep recordings took place between 0900 and 1200 hours (light onset at 0900 hours). Cortical EEG and EMG signals were amplified (Grass Technologies) and digitized at 256 Hz using a sleep recording system (VitalRecorder, Kissei Comtec). The signals were digitally filtered and spectrally analyzed by fast Fourier transform and polysomnographic recordings were visually scored by 4-s epochs for wake, non-rapid eye movement (non-REM) and REM sleep. Microarousals consisted of a single epoch (≤4 s event) scored as a short waking event characterized by rapid EEG desynchronization with concomitant EMG activation. Polysomnographic scoring was performed blindly to the identity of the mice.

### Novel environment test

Hcrt::ChR2–mCherry and control mice were subjected to 1 h, 20 Hz (10 s IPI) photostimulation in their home cage. At the end of the photostimulation session, mice were placed in a novel white-walled open field arena (75 × 75 × 37 cm). Mice were introduced in a delimited centre zone (22 × 22 cm) and were given the opportunity to explore the arena for 10 min while being tracked using the real-time automated ViewPoint VideoTrack system. Time spent in the different zones (centre and periphery), inactive time, velocity and total distance travelled were recorded. At the conclusion of each trial the surface of the arena was cleaned with 70% ethanol.

### Chronic FR

Mice received a limited amount of food at the end of the daily light phase during 7 days. Each evening, the mice were weighted and the amount of food was adjusted to reach ~85% of their initial body weight. On day 8, blood was collected to measure corticosterone concentrations for FR baseline or in response to 20 Hz photostimulation for 1 h, 10 s IPI in Hcrt::ChR2–mCherry and Hcrt::mCherry mice (randomized measures; Fig. 3a), or after 7 consecutive days of local LHA leptin versus PBS treatment in LepRb::eYFP mice (Fig. 3d). LepRb-cre mice were transduced with DIO–eYFP AAV virus within the LHA 3 weeks prior to the experimental procedures to visualize LepRb-expressing neurons for histology. LHA LepRb::eYFP mice were then killed to label and quantify c-Fos/Hcrt- and pSTAT3-expressing cells in response to chronic FR in leptin- versus PBS-treated mice.

### Restraint stress

LHA LepRb::ChR2–eYFP and control LepRb::eYFP mice were subjected to 30-min immobilization stress by keeping them in a well-ventilated mouse restrainer device (Fisher Scientific). Paired photostimulation was applied at 10 Hz continuously during the 30 min restraint (Fig. 5b). Blood was collected 20 min after the end of stress for corticosterone measurement and mice were killed 90 min later to assess stress-related c-Fos/Hcrt staining and quantification.

### Pharmacology

Recombinant murine leptin (National Hormone and Peptide Program (NHPP), Harbor-UCLA Medical Center, Torrance, CA, USA) was injected 15 min prior to the experimental procedures. Mice were briefly restrained to remove optic fibres and insert a 33-gauge bilateral needle (Plastics One) into the implanted cannula guide. Needle is connected with a tube to a 0.5 μl Hamilton syringe plugged into a programmable pump (World Precision Instruments) infusing either leptin (0.5 μg, 0.1 μl) or PBS (0.1 μl, pH 8) on each side of the LHA over 1 min. Cannula was left in place for 2 min after injection and then removed. Optical fibres in Hcrt::ChR2–mCherry and control mice were re-inserted at the end of the injection. This procedure required daily mild restraint of the animals, which raised baseline corticosterone levels and potentially induced additive stress along with the effects of chronic FR. However, both the experimental and control mice were subjected to the same manipulation.

Hcrt-1/Orex-A peptide (Bachem) was dissolved in saline (3 nmol, 1 μl) and injected into the anterior hypothalamus aiming for the PVN area (AP 1.3 mm, ML 0.3 mm from the bregma; DV 4.5 mm) 30 min prior to blood collection at the corresponding circadian time of photostimulation sessions.

The Hcrt antagonist SB-334867 (lot #1960, Tocris bioscience) was dissolved in 1% DMSO/10% cyclodextrine (Sigma) sterile water vehicle and administered i.p. at the dose of 30 mg kg^−1^ 30 min before photostimulation.

All drug tests were performed randomly with either vehicle or light/no light conditions in both genotypes.

### Cardiovascular monitoring

We monitored heart rate (beats per minute) and pulse distention (μm), which is a measurement of blood flow volume proportional to blood pressure, through a mouse infrared sensor collar clip (MouseOx, Starr Life Sciences) for non-invasive and non-stressful measurement. To avoid stress elicited by restraint in conscious mice that may occlude the effects of Hcrt photostimulation, we performed hemodynamic measurements in anaesthetised Hcrt::ChR2–mCherry and Hcrt::mCherry mice placed under isoflurane (1.5%; oxygen 1 l min^−1^), which maintains stable mean arterial pressure and heart rate, comparable to those observed in the animal’s conscious state without altering corticosterone secretion. We monitored heart rate and pulse distention pre-stimulation baseline for 10 min until it stabilized. Then mice received 20 Hz photostimulation with 10 s IPI for 15 min and we recorded post-stimulation response for 10 min.

### Viral injections for slice electrophysiology

We employed two strategies for specifically expressing ChR2 in LHA LepRb neurons while visualizing Hcrt neurons for slice electrophysiology: (1) LepRb::cre mice were injected with a mixture of two viruses, AAV5–DIO–ChR2–eYFP and Lenti-Hcrt::mCherry (1 μl); (2) Bi-transgenic LepRb::cre/Hcrt::eGFP mice were injected with AAV5–DIO–ChR2–mCherry (0.8 μl). All injections were performed on 4–6-week-old mice of either sex and unilateral (AP 1.4 mm, ML 0.8 mm, two DV points: 5.1 and 4.95 mm) at a rate of 0.05 μl min^−1^. Following 3–4 weeks, the expression of ChR2–eYFP or ChR2–mCherry in LepRb neurons was consistently concentrated within the LHA, but sometimes impinged on neighbouring hypothalamic regions. The results of these two strategies were qualitatively similar and data were pooled.

### Brain slice preparation and recording

Coronal hypothalamic slices containing the LHA (225 μM) were prepared from 2–3.5-month-old transgenic mice, at least 3–4 weeks following virus injection. Mice were deeply anaesthetised with ketamine/xylazine before being transcardially perfused with an ice-cold, high-sucrose cutting solution consisting of: 87 mM NaCl, 1.25 mM NaH2PO4, 25 mM NaHCO3, 2.5 mM KCl, 0.5 mM CaCl2, 7 mM MgCl2, 25 mM glucose and 75 mM sucrose, saturated with 95% O2/5% CO2, prior to slicing. Hypothalamic slices containing the LHA were then placed in a recovery chamber containing artificial cerebrospinal fluid (CSF), consisting of : 125 mM NaCl, 2.5 mM KCl, 25 mM NaHCO3, 1.25 mM Na2PO4, 25 mM glucose, 2 mM CaCl2 and 1 mM MgCl2, saturated with 95% O2/5% CO2 and allowed to recover prior to recording. Whole-cell patch-clamp recordings from fluorescent LHA neurons were carried out using thin-walled borosilicate glass pipettes (World Precision Instruments) with pipette resistance values of 2–4 MΩ. For whole-cell current-clamp and voltage-clamp recordings of identified LepRb-expressing neurons, pipettes were filled with an internal solution consisting of: 135 mM potassium methanesulfonate, 10 mM NaCl, 2 mM MgCl2, 1 mM EGTA, 10 mM HEPES, 14 mMphosphocreatine, 4 mM Mg-ATP and 0.3 mM Na-GTP (pH 7.3). In addition, voltage-clamp recordings from LepRb-expressing neurons were carried out at −60 mV in the presence of TTX (500 nM). For whole-cell voltage-clamp recordings from identified Hcrt neurons, pipettes were filled with a high-Cl internal solution consisting of: 135 mM CsCl, 10 mM HEPES, 10 mM EGTA, 2 mM NaCl, 4 mM Mg-ATP, 5 mM TEA-Cl, 1 mM QX-314-Cl and 0.1 mM spermine (pH 7.3). These recordings were carried out at −60 mV in the presence of D-APV (50 μM) and NBQX (10 μM). All recordings were conducted using a Multiclamp 700B amplifier (Molecular Devices) filtered at 2 kHz and digitized at 10 kHz using a National Instruments digitizer and were acquired using custom procedures in IgorPro (Wavemetrics). Photostimulation was carried out using a TTL-controlled ultra-high power white LED (Prizmatix), passing through an enhanced green fluorescent protein (EGFP) filter cube and controlled using a Master-8 Pulse Generator (A.M.P.I.). Intensity of blue-light photostimulation was ~0.9 mW. Pulse duration was 1–5 ms for characterization of photostimulation-induced firing fidelity and 5 ms for evaluation of synaptic release. All recordings were carried out at room temperature (RT; 21–22 °C).

### Immunohistochemistry

Brains were postfixed overnight (ON) in 1 × PBS solution (pH 7.4) containing 4% paraformaldehyde, and cryoprotected in 30% sucrose for 24 h. Each brain was cut in coronal serial sections 40 μm thick on a cryostat (Leica Microsystems).

For double-labelled brightfield immunohistochemistry experiments (Fig. 1g, Supplementary Fig. 5a), free-floating sections were treated with 1% H_2_O_2_ in PBS to inactivate endogenous peroxidase activity. Non-specific antigens were blocked by incubating in PBS containing 2% normal donkey serum (NDS) and 0.1% Triton X-100 for 2 h at RT. Sections were then incubated ON at RT with the primary polyclonal rabbit anti-c-Fos (1:2,000; #PC05; Calbiochem). After several rinses in PBS supplemented with 0.1% Triton X-100 (PBST), sections were incubated for 2 h in the blocking buffer with biotinylated anti-rabbit secondary antibody (1:200 dilution with ABC Vectastain Elite kit, Vector Laboratories, Burlingame, CA, USA) at RT. Then, further rinses were followed by incubation in avidin–biotin–horseradish peroxidase solution (ABC Vectastain Elite kit) for 1 h. c-Fos IR neurons were dyed in black/grey with 3,3′-diaminobenzidine-nickel-enhanced technique^43^. Sections were washed ON in PBS at 4 °C and processed for the second procedure to label Hcrt neurons. This procedure was similar to the first one except that: (1) the primary antibody was the anti-Hcrt-1/orexin-A (C-19) (1:500; sc-8070; batch B0309; Santa Cruz Biotechnology) polyclonal goat antibody and (2) sections were finally dyed in brown using 0.04% diaminobenzidine only. Sections were mounted onto SuperFrost slides (Fischer Scientific), dehydrated in graded alcohols, cleared in xylene and coverslipped with DPX mounting medium (BDH Chemicals).

For immunofluorescent experiments in LepRb::ChR2–eYFP or LepRb::eYFP mice (Figs 3f,h and 5d, Supplementary Fig. 5), brain sections were washed in PBST for 10 min at RT and incubated in blocking solution for 1 h at RT. To assess pSTAT3 immunostaining, sections were permeabilized with methanol (10 min at −20 °C) and rinsed in PBS for 5 min. Sections were incubated in either primary rabbit anti-c-Fos (1:2,000) or rabbit anti-pSTAT3 (1:100; #9145, Cell Signaling technology) antibodies with the primary goat anti-Hcrt (1:500) antibody in the blocking solution ON at RT. After 3 × 10 min washes in PBST, sections were incubated with Alexa Fluor 594 nm donkey anti-rabbit IgG (1:250, Molecular Probes) and Alexa Fluor 350 nm donkey anti-goat IgG (1:250, Molecular Probes) in blocking solution for 2 h at RT. Sections were washed 5 × 10 min in PBS, mounted onto SuperFrost slides and coverslipped with Vectashield hardset mounting medium (Vector Laboratories).

pSTAT3 expression was analyzed in LepRb::cre mice injected beforehand with AAV–DIO::eYFP viral vector in the LHA to visualize LepRb–eYFP neurons. These mice were subjected to 7 days of FR and chronic local injections of either leptin or PBS into the LHA.

Photomicrographs were taken from representative histological examinations conducted at the end of the experiments over all experimental mice from the different genotypes and treatment sub-groups. Counting was performed by an investigator (P.B.) blind to the identity of the experimental mice (Hcrt::ChR2–mCherry or Hcrt::mCherry; leptin- or PBS-treated mice; LepRb::ChR2–eYFP or LepRb::eYFP). For Hcrt-induced c-Fos expression, all structures (PVT, PVN, DR and LC) were analyzed in a 1:3 series of five rostro-caudal sections per animal corrected over 450 μm in each area for control and ChR2 mice. PVN and LC sections were quantified bilaterally.

### Statistics

All data were analyzed using Prism 6.0 (GraphPad Software). Statistics were first performed with two-way repeated measures ANOVA to test the significance between virus (ChR2 or mCherry/eYFP control) and photostimulation conditions, stress/fed states (no restraint or restraint stress; *ad libitum* food or FR) and leptin-treated conditions. Repeated measures one-way ANOVA was used to test for the effects of photostimulation (pre, stim, post; Fig. 1b, Fig. 2a–d, Fig. 5g) at various durations (Fig. 1d), under fed or FR state (Fig. 3a), associated with leptin pharmacological treatments (Fig. 3b) or under restraint stress (Fig. 5e) within a virus group (ChR2 or control) followed by Tukey *post-hoc* tests and two-tailed unpaired Student’s *t-*test between virus groups, presented on the figures. All electrophysiological data are presented as mean±s.e.m. For *t-*tests, equality of variances was analyzed with an *F*-test and Welch's correction was employed when variances of populations was significantly different.

Analyses of data shown in Fig. 1b–h, Fig. 2a–i, Fig. 3a–c, Fig. 5c–h and Supplementary Figs 1–4 were carried out with the investigator blinded to the genotypes. In the remaining experiments, no blinding was performed.

## Additional information

**How to cite this article**: Bonnavion, P. *et al.* Antagonistic interplay between hypocretin and leptin in the lateral hypothalamus regulates stress responses. *Nat. Commun.* 6:6266 doi: 10.1038/ncomms7266(2015).

## Supplementary Material

Supplementary Information Supplementary Figures 1-4
