# Supplementary material for: Antagonistic interplay between hypocretin and leptin in the lateral hypothalamus regulates stress responses
Source: Nat Commun. 2015 Feb 19;6:6266. doi: 10.1038/ncomms7266 (PMC4335349; doi:10.1038/ncomms7266)
Supplement: Supplementary Information — Supplementary Figures 1-4 [file ncomms7266-s1.pdf]

Supplementary Figure 1

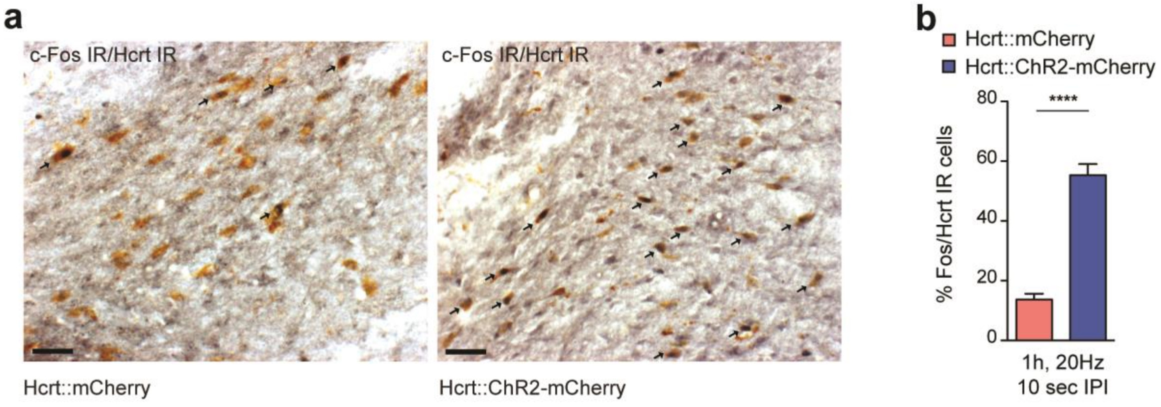

(**a–b**) *In vivo* bilateral photostimulation in Hcrt::ChR2-mCherry mice selectively activates Hcrt neurons. (**a**) Representative photomicrographs of LHA sections co-stained for c-Fos (black) and Hcrt (brown) from Hcrt::mCherry (n = 7) and Hcrt::ChR2-mCherry (n = 7) mice. Black arrows indicate dual-labeled neurons with both anti -Hcrt and -cFos antibodies. Scale bar: 50 µm. (**b**) Bar graphs quantify the percentage of Hcrt IR neurons expressing c-Fos after 1h photostimulation at 20Hz, 10 s IPI, in Hcrt::mCherry control mice (red, n = 7) and Hcrt::ChR2-mCherry mice (blue, n = 7). $^{****}P < 0.0001$ two-tailed unpaired Student's t test between transduced mice. Data represent means ± s.e.m.

Supplementary Figure 2

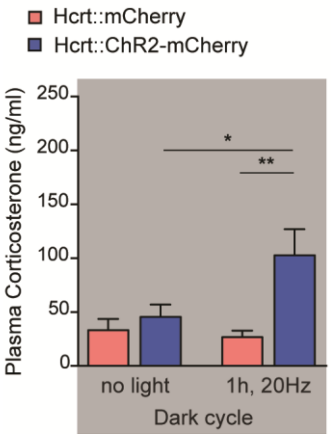

Hcrt-evoked increase in plasma corticosterone levels is circadian-independent. *In vivo* bilateral photostimulation of Hcrt neurons in Hcrt::ChR2-mCherry mice (blue, n = 10) evoked a significant elevation of plasma corticosterone, but not in Hcrt::mCherry control mice (red, n = 11). $^{**}P < 0.01$, two-way repeated measures ANOVA between viral transduction and stimulation condition; $^{*}P < 0.05$, $^{**}P < 0.01$, two-tailed Student's t test.

Supplementary Figure 3

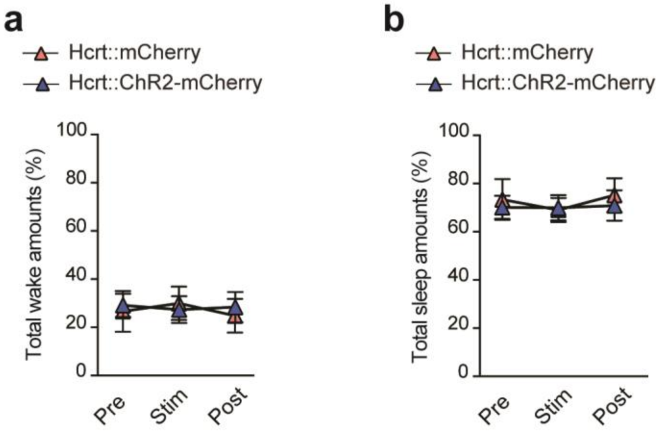

(**a–b**) Consequences of persistent activation of Hcrt neurons on wake and sleep quantities. Total amount of wake **(a)** and total amount of total sleep (Non-REM and REM sleep, **(b)**) in % of total time recorded in Hcrt::mCherry (red, n = 7) and Hcrt::ChR2-mCherry mice (blue, n = 9) averaged over 1h before (Pre), during (Stim) and after (Post) 20Hz, 10 s IPI photostimulation of Hcrt neurons. While stimulated ChR2 mice exhibit higher sleep fragmentation with increased sleep-to-wake transitions and microarousal events (Fig. 2a,b), total wake and sleep quantities were unchanged compare with baseline and control animals. P = 0.50 (**a**), P = 0.99 (**b**), two-way repeated measures ANOVA between viral transduction and stimulation condition; P = 0.94 (**a**), P = 0.97 (**b**), repeated measures one-way ANOVA followed by Tukey's *post-hoc* test and two-tailed Student's t test between transduced mice.

Supplementary Figure 4

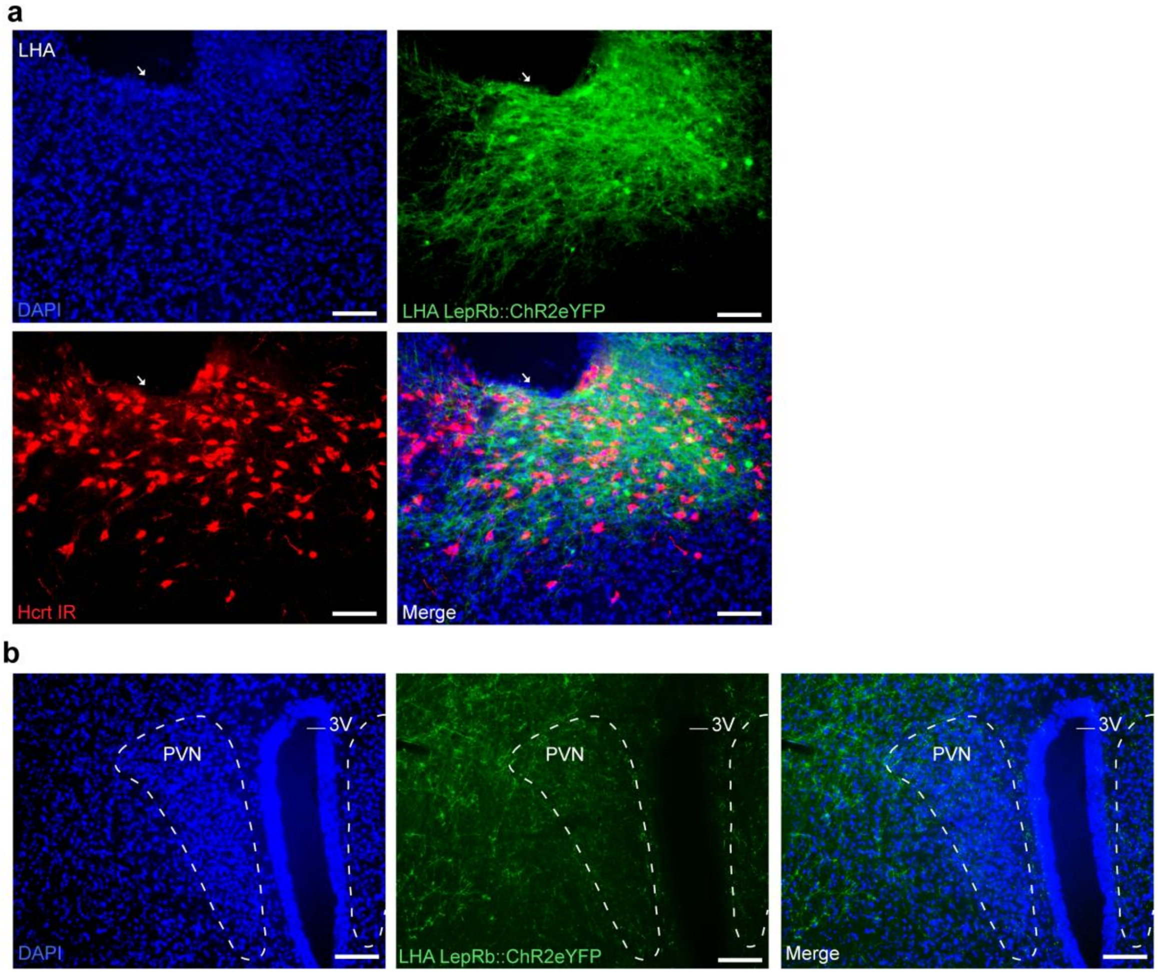

(**a**–**b**) Virus diffusion and transgenes expression in the LHA and PVN region of LepRb::cre mice. (**a**) Representative photomicrographs illustrating viral ChR2-eYFP expression (n = 9) in LHA LepRb::cre neurons (green) directly below fiber track intermingled with Hcrt neurons labeled with an anti-Hcrt antibody (red) and counterstained with DAPI (blue). White arrows show the location and tip of the optical fiber. Scale bar: 100 µm. (**b**) Representative photomicrographs of LHA LepRb::ChR2-eYFP neurons projections to the PVN region (green) counterstained with DAPI (blue). Some ChR2-eYFP terminals are found in the anterior nucleus of the hypothalamus but not within the PVN (n = 9). White dotted lines delineate PVN. Scale bar: 100 µm.
